# Directed evolution of anti-HER2 DARPins by SNAP display reveals stability/function trade-offs in the selection process

**DOI:** 10.1093/protein/gzv029

**Published:** 2015-06-30

**Authors:** Gillian Houlihan, Pietro Gatti-Lafranconi, David Lowe, Florian Hollfelder

**Affiliations:** 1 Department of Biochemistry, University of Cambridge, 80 Tennis Court Road, Cambridge CB2 1GA, UK; 2 MedImmune Ltd, Milstein Building, Granta Park, Cambridge CB1 6GH, UK

**Keywords:** alternative scaffold, antibody, DARPin, directed evolution, *in vitro* compartmentalisation, SNAP display, trade-off

## Abstract

*In vitro* display technologies have proved to be powerful tools for obtaining high-affinity protein binders. We recently described SNAP display, an entirely *in vitro* DNA display system that uses the SNAP-tag to link protein with its encoding DNA in water-in-oil emulsions. Here, we apply SNAP display for the affinity maturation of a designed ankyrin repeat proteins (DARPin) that binds to the extracellular domain of HER2 previously isolated by ribosome display. After four SNAP display selection cycles, proteins that bound specifically to HER2 *in vitro*, with dissociation constants in the low- to sub-nanomolar range, were isolated. *In vitro* affinities of the panel of evolved DARPins directly correlated with the fluorescence intensities of evolved DARPins bound to HER2 on a breast cancer cell line. A stability trade-off is observed as the most improved DARPins have decreased thermostability, when compared with the parent DARPin used as a starting point for affinity maturation. Dissection of the framework mutations of the highest affinity variant, DARPin F1, shows that functionally destabilising and compensatory mutations accumulated throughout the four rounds of evolution.

## Introduction

Screening and selection technologies have been fundamental for the isolation of antibodies and other binding proteins (Hoogenboom, 2005; Leemhuis *et al.*, 2005; Bradbury *et al.*, 2011; Colwill and Graslund, 2011). The availability of high-affinity binding molecules with exquisite target specificity has led to their widespread use in diagnostics (Schellekens *et al.*, 2000) and therapeutics (Chan and Carter, 2010). Next-generation antibodies including antibody-drug conjugates, biosuperiors and multispecific antibodies hold promise for greater clinical efficacy (Beck, 2011; Buss *et al.*, 2012; Evans and Syed, 2014). However, antibody fragments often have limited stability, a tendency to aggregate and require an oxidising environment for structurally critical disulfide bonds to form, therefore complicating recombinant expression (Lowe *et al.*, 2011). Binding scaffolds with superior biophysical properties have been engineered as alternatives for molecular recognition (Binz *et al.*, 2003; Gebauer and Skerra, 2009). These alternatives include designed ankyrin repeat proteins (DARPins), designed leucine-rich repeat proteins with varying curvatures (Ramisch *et al.*, 2014), affibodies (Nord *et al.*, 1997; Boersma and Plückthun, 2011), monobodies (Koide *et al.*, 1998), cystine-knot miniproteins (Kolmar, 2009) and anticalins (Beste *et al.*, 1999). Such scaffolds consist of a characteristic framework that presents surface loops with randomised regions, setting up potential binding interfaces to interact with different target antigens.

One class of alternative scaffolds, DARPins, are derived from the ankyrin repeat motif, which modulates a plethora of binding interactions in bacteria, *archaea* and *eukarya* (Mosavi *et al.*, 2004). Repeats consist of a β-turn followed by two antiparallel α-helices and a loop connecting the next helix. A consensus design approach based on sequence and structural alignments of natural ankyrin repeat proteins was adopted to design modules consisting of conserved framework positions and randomised potential target interaction positions at the binding interface. The designed DARPins were well-expressed proteins with high thermodynamic stabilities. DARPin libraries with different numbers of internal repeated modules were constructed with flanking charged N- and C-terminal caps to shield the hydrophobic core (Binz *et al.*, 2003). High-affinity binders for a number of globular proteins have been successfully isolated by both ribosome and phage display (Binz *et al.*, 2004; Sennhauser *et al.*, 2007; Steiner *et al.*, 2008).

**Fig. 1:**
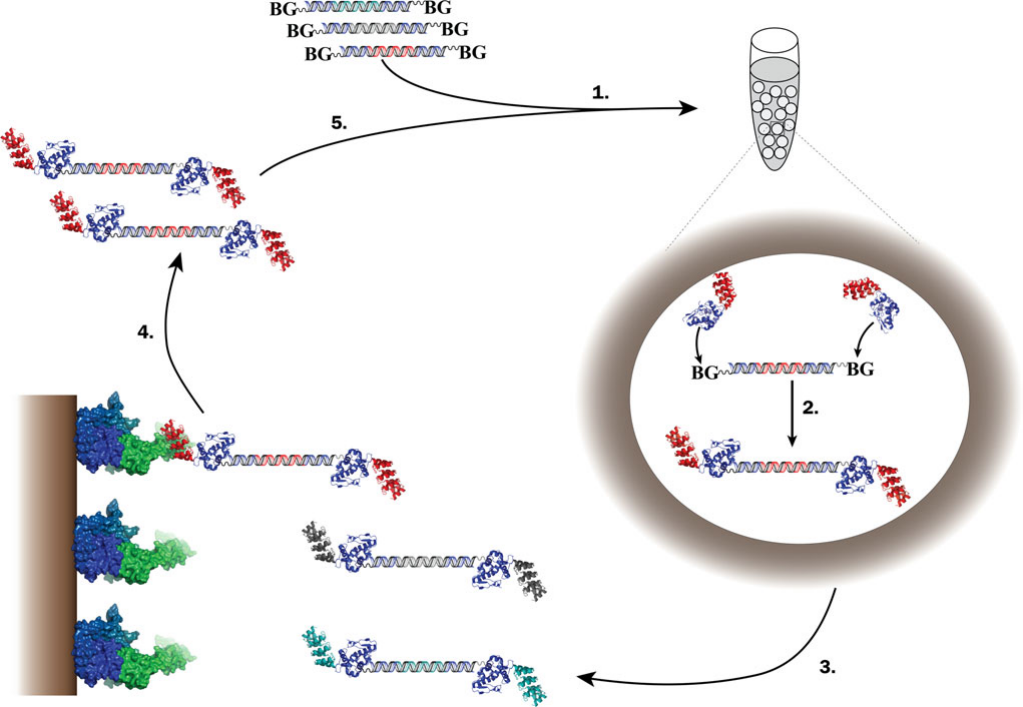
SNAP display selection scheme. (1) Water-in-oil emulsion droplets compartmentalise DNA library members separately, such that one gene (or none) is Poisson-distributed in droplets. *In vitro* expression is performed from single linear DNA templates. (2) The SNAP-tag forms a covalent thioether bond between the protein-coding DNA (bearing a covalently-linked SNAP-substrate, benzylguanine, BG) and the corresponding expressed protein (Keppler *et al.*, 2003). The stability of the covalent genotype–phenotype linkage enables panning selections (3) to be performed under a wider range of conditions without the risk of disassembly of genotype and phenotype. Panning conditions are tuned to achieve the required level of stringency for the isolation of binders (4). The DNA recovered from selections is amplified by PCR (5). After each round of selection, additional mutations are easily introduced during PCR by low fidelity polymerases. Reproduced with permission from Houlihan *et al.* (2014).

While all panning selections from display libraries are carried out *in vitro*, the display technology may have an influence on the selection outcome, because the quality of the display is correlated to the available genetic diversity that can be explored. For example, translocation in phage display and ternary complex stability in ribosome display are requirements for successful display, and can bias a selection campaign by restricting the level of diversity that can be screened. To assess the utility of an alternative technology, we now apply SNAP display (Stein *et al.*, 2007; Kaltenbach and Hollfelder, 2012; Houlihan *et al.*, 2014) for the isolation of DARPins binding the HER2/neu transmembrane tyrosine kinase receptor. SNAP display is a cell-free DNA display system, which uses water-in-oil droplets to compartmentalise single DNA library members (Fig. 1) (Stein *et al.*, 2007; Schaerli and Hollfelder, 2009; Houlihan *et al.*, 2013). A covalent linkage is created in emulsion droplets by chemical reaction of an *in vitro*-expressed O^6^-alkylguanine DNA alkyltransferase (AGT)-DARPin fusion with BG-labelled DNA templates (Juillerat *et al.*, 2003; Keppler *et al.*, 2003). SNAP display exists in several formats, which, for example, take advantage of avidity during selection (Kaltenbach *et al.*, 2011) or enable quantitative binding selections based on fluorescence detection of megavalent constructs [BeSD, see (Diamante *et al.*, 2013)]. The covalent nature of the SNAP display construct yields the DNA of each binding hit even under panning conditions that would lead to disintegration of non-covalent constructs. The stability of DNA (rather than RNA) should also facilitate robust library recovery during selection. Other display technologies including ribosome (Zahnd *et al.*, 2007a,b; Douthwaite and Jackson, 2012; Dreier and Plückthun, 2012; Plückthun, 2012), mRNA (Keefe and Szostak, 2001), DNA (Doi and Yanagawa, 1999) and mHaeIII (Bertschinger *et al.*, 2007) display have already been previously employed in directed evolution campaigns, while library selections by SNAP display remain to be shown.

Here, we report the first successful application of SNAP display for the *in vitro* evolution of DARPins targeting the extracellular domain of HER2. The target protein, HER2, is involved in regulating cell growth, survival, adhesion and differentiation (Yarden, 2001) and found to be overexpressed on the tumour cell surface in ∼20% of all human breast cancers (Slamon *et al.*, 1987), which results in an aggressive phenotype with a poor clinical outcome for patients (Winstanley *et al.*, 1991). Trastuzumab (Herceptin), a monoclonal antibody that binds to domain IV of HER2, has an antiproliferative effect on cells overexpressing HER2 and is currently used as a treatment for breast cancer (Slamon *et al.*, 2001). Despite the clinical success of Trastuzumab, a significant proportion of patients acquires resistance within 1 year of treatment (Albanell and Baselga, 2001; Montemurro *et al.*, 2006). Hence, there is a strong argument to develop new binders and strategies to find alternative ways of overcoming resistance (Schrama *et al.*, 2006).

Starting from DARPin G3-HAVD (Zahnd *et al.*, 2006), which binds with sub-micromolar affinity to the extracellular domain of HER2, affinity maturation was performed by SNAP display. Starting with libraries of >10^9^ variants, four rounds of SNAP display selection cycles yielded binders with dissociation constants for the extracellular domain of HER2 in the low- to sub-nanomolar range, and that also bind to the HER2 receptor on cells. The most improved variant, DARPin F1, accumulated nine mutations throughout the maturation process and had an almost 700-fold increase in affinity compared with its parent. We observe a stability trade-off as the most improved DARPins have decreased thermostability, when compared with the parent DARPin used as a starting point for affinity maturation.

## Materials and methods

### Error-prone polymerase chain reaction

The pQE30 plasmid containing the gene encoding DARPin G3-HAVD was used as a template for error-prone polymerase chain reaction (PCR) using the GeneMorph II random mutagenesis kit (Agilent Technologies). The PCR was performed under conditions that maintained a low mutation rate (0–4 mutations per gene; according to the manufacturer's recommendations) using a forward primer (5′-TTGGGAGGTACCGGCGGTCTG-3′) containing a KpnI site and reverse primer (5′-GTTAGCAGCCGGATCCTCACTATAAC-3′) containing a BamHI site. Briefly, a 50 µl reaction contained template DNA (1 µg), dNTPs (200 µM), forward and reverse primers (2 µM), Mutazyme II reaction buffer (1×) and Mutazyme II DNA polymerase (2.5 U). Thermal cycling consisted of an initial heat activation step for 2 min at 95°C followed by 30 cycles of denaturation at 95°C (30 s), annealing at 54°C (1 min) and extension at 72°C (1 min) followed by a final elongation step at 72°C (10 min). The PCR product was digested with DpnI (NEB) to remove any remaining plasmid and purified using the QIAquick PCR purification kit (QIAgen). A portion of the library was used for reassembly (see below), while another portion was cloned into pIVEX to determine the mutation rate by sequencing 15 randomly picked colonies.

### DARPin library construction

In separate PCR steps (see Supplementary Fig. S1A), the assembly fragments (5′ untranslated region-AGT and 3′ untranslated region) were amplified with primers 5′-UTR fwd (5′-CCGCCGGTACCTCCCAAGCCTG-3′), 5′-UTR rev (5′-TGGCGAAAGGGGGATGTGC-3′), 3′ UTR fwd (5′-AGTGAGGATCCGGCTGCTAACAAAGCC-3′) and 3′ UTR rev (5′-TGCTAGCGCTATATGCGTTGATGC-3′), respectively, from the plasmid pIVEX-AGT-DHFR using Pfu Ultra II polymerase (Agilent Technologies). Identical thermo-cycling conditions were used as above apart from the annealing temperatures (60°C). After DpnI digestion and purification of the amplicons, the library was assembled by overlap extension PCR using the PCR fragments from the separate amplification steps described above using the primers LMB 2-6 (5′-ATGTGCTGCAAGGCGATTAAG-3′) and pIV-BG (5′-BG-­GCGTTGATGCAATTTCTATGC-3′). The final construct was subjected to PEG-MgCl_2_ precipitation, as described in Stein and Hollfelder (2009) to remove any remaining primer-dimers. Samples were run on an agarose gel to confirm DNA fragments with the correct size were amplified (see Supplementary Fig. S1B).

### Affinity selections using SNAP display

*In vitro* transcription and translation were performed as previously described (Houlihan *et al.*, 2014). Briefly, 5 ng of DNA were added to the *in vitro* transcription and translation (IVTT) mix [PURExpress, NEB (Kanamori *et al.*, 2014)], then homogenised in ice-cold mineral oil mix [95% mineral oil (Sigma-Aldrich), 4.5% Span 80 (w/w) (Fluka), 0.5% Tween 80 (w/w) (Sigma)]. The emulsion was incubated at 25°C for 4 h, then the aqueous phase extracted in recovery and binding buffer [phosphate buffered saline (PBS) supplemented with 5 mM EDTA and 10 μM BG]. The displayed protein library was incubated with biotinylated HER2 (purchased from R&D Systems, cat. no. 1129-ER-050) and captured on streptavidin-coated beads (Dynal). Biotinylation of HER2 was performed as described in Houlihan *et al.* (2014). After incubation for 1 h, streptavidin beads were washed (five times with PBS supplemented with Tween 20, 0.01% v/v) and remaining HER2-bound DARPins were eluted with KOH (6 mM). Recovered DNA was amplified using the primers sel-fwd (5′-TTGGGAGGTACCGGCGGTCTG-3′) and sel-rev (5′-GTTAGCAGCCGGATCCTCACTATAAC-3′) and reassembled as described above. DNA was purified using QIAquick PCR purification kit (QIAgen), precipitated with PEG-MgCl_2_ and used in the following round of selection or cloned into pQE30 for sequencing.

### qPCR of selection outputs

DNA was quantified using a Rotor-Gene 6000 machine (Corbett Research). Three microlitres of a selection output were used in a real-time mix consisting of 1× Sensimix SYBR no-rox (Bioline) and primers Fwd-DARPin-50 (5′-AGGCTTGGGAGGTACCG-3′) and DARPin-rtpcr-rev (5′-GCTAAGTGAAGAGGGGTTAG-3′). Cycling conditions were as follows: 40 cycles of denaturation at 95°C for 15 s, annealing at 55°C for 15 s and extension at 72°C for 20 s.

### Site-directed mutagenesis of DARPin F1 to revert mutated residues

DARPin F1 was mutated using non-overlapping oligonucleotides that contained the desired mutation during whole plasmid PCR. The amplified DNA was digested with DpnI, treated with T4 PNK, ligated into pQE30 using the restriction enzymes BamHI and HindIII and transformed into M15 cells for protein expression. DARPin F1 mutants were expressed and purified as described above.

### Protein expression and purification

SNAP display-selected DARPins were cloned into the plasmid pQE30 (QIAgen) downstream of a hexa-histidine tag using primers DARPin-1 (5′-ACGTACGATCCGATCTAGGCAAGAAACTACTTGAGGC-3′) and DARPin-2 (5′-AATTAAGCTTTCACTATAACTTTTGGAGAATTTCAGCCAG-3′). Clones were transformed and expressed in the bacterial strain, M15. For small-scale expression, overnight cultures were added to 10 ml LB media and grown until an OD_600_ of 0.6 was reached. Protein expression was induced with 1 mM IPTG and cells were grown at 37°C for 4 h. Cells were centrifuged at 6000*g* for 10 min at 4°C. Pellets were resuspended in lysis buffer (1× bugbuster, 40 mM imidazole, 1× PBS) and purified using His trap spin columns (GE healthcare), which yielded >90% pure protein. For large-scale purification, DARPins were expressed on a 1 l scale and purified using affinity chromatography (HisTrap ff crude columns - GE healthcare). DARPins were further purified with a size-exclusion chromatography step using a Superdex 75 column (GE healthcare).

For competition experiments, DARPin H10-2-G3 was cloned into the plasmid pASK-IBA5plus downstream of a Strep-Tactin tag using BamHI and HindIII cloning sites. Top 10 *E.coli* cells were transformed with the plasmid and a single colony was grown overnight at 37°C in LB media. The LB media (10 ml) was inoculated with overnight cultures and grown until an OD_600_ of 0.6 was reached and induced with 0.2 μg/ml anhydrotetracycline. The cells were harvested by centrifugation and resuspended in buffer W (100 mM Tris–HCl, pH 8.0, containing 150 mM NaCl and 1 mM EDTA). Strep-tagged DARPin H10-2-G3 was purified using Strep-Tactin spin columns (IBA Life sciences) according to the manufacturer's instructions.

### ELISA (crude and soluble)

For ELISA screening, selected DARPins from SNAP display outputs were amplified by PCR using the primers DARPin-1 (5′-ACGTACGATCCGATCTAGGCAAGAAACTACTTGAGGC-3′) and DARPin-2 (5′-AATTAAGCTTTCACTATAACTTTTGGAGAATTTCAGCCAG-3′), cloned into the plasmid pQE30 (QIAgen) and used to the *E. coli* strain M15. Individual colonies were picked and grown in a 96-deep well plate in 200 μl of LB medium overnight at 37°C. After addition of 1.3 ml media, cells were grown until an OD_600_ of 0.6 was reached. Protein expression was induced with IPTG (1 mM final concentration) and cells were grown for a further 4 h at 37°C. Cells were harvested by centrifugation at 6000*g* for 10 min at 4°C. Cell pellets were lysed with 50 μl 1× Bugbuster (Merck-Millipore) per well and centrifuged to pellet any remaining cellular debris. The crude extract was diluted with 450 μl PBS, 100 μl was added to a streptavidin-coated 96-well plate (Thermo scientific) previously coated with biotinylated HER2 and incubated for 1 h at room temperature. All wells were washed five times with PBS supplemented with Tween 20 (0.01%). An RGS-anti-His antibody conjugated to horseradish peroxidase (HRP) (QIAgen) was diluted 1:2000 in blocking buffer (QIAgen) and 50 μl added to each well. Colorimetric detection was done by the addition of 50 μl of the substrate, 3,3′,5,5′-tetramethylbenzidine (Sigma) and the reaction was stopped with 50 μl of 0.5 M H_2_SO_4_.

For soluble ELISAs, the procedure outlined above was performed using 10 μg/ml of purified DARPin in place of crude extract. For specificity ELISAs, the same procedure as soluble ELISAs were performed but streptavidin wells were coated with different antigens. For competition ELISA with DARPin H10-2-G3 (strep-tag), the evolved DARPins (His-tagged) were combined with 150 nM DARPin H10-2-G3 and the level of HER2-bound DARPin (His-tagged) was measured as above.

### Bio-layer interferometry

*K*_D_ values were determined by bio-layer interferometry using an Octet Red instrument (ForteBio, Inc.). Biotinylated HER2 (10 μg/ml) in 1× kinetics buffer (PBS, pH 7.4, 0.01% bovine serum albumin (BSA) and 0.01% Tween 20) was loaded onto streptavidin-coated biosensors (ForteBio, Inc., 18-5020) and incubated with DARPins in kinetics buffer. A titration of eight different DARPin concentrations was used to measure kinetics with the highest concentration starting from 50 nM. Each measurement consisted of five steps: baseline acquisition, 60 s; HER2 loading onto SA sensor, 300 s; baseline acquisition, 60 s; association of DARPin, 300 s; dissociation of DARPin, 300 s. Baseline and dissociation steps were performed in kinetics buffer. All steps were performed with sample agitation at 1000 rpm. Binding kinetics were determined using a 1:1 Langmuir-binding model in kinetics data analysis mode using the Fortebio data processing software.

### Tissue culture and flow cytometry

The breast cancer cell line SK-BR-3 (ATCC) was cultured in McCoy's 5a modified medium supplemented with 10% foetal bovine serum and grown in 5% CO_2_ at 37°C. The cells were dissociated from the flask, centrifuged at 1200*g* for 5 min at 4°C and the pellet resuspended in sterile PBS. Cells were transferred to 96-well tissue culture plates (5 × 10^6^ cells per well) and stained with LIVE/DEAD Fixable Aqua Stain (Invitrogen) (100 μl/well) for 30 min protected from light. Cells were washed with flow cytometry staining (FCS) buffer and centrifuged at 1200*g* for 5 min. DARPins and controls were added to the cells (1 µg/ml) and incubated for 1 h at room temperature. The cells were centrifuged at 1200*g* for 5 min, pellets were resuspended in 100 μl of an anti-histidine tagged antibody (Millipore 05-949) (5 μg/ml) and incubated for 60 min in the dark. After washing in FCS buffer, the cells were resuspended with a goat anti-mouse R-phycoerythrin-conjugated secondary antibody (Jackson Immunoresearch 115-116-146) and incubated for 60 min in the dark. Trastuzumab- and Cat002-treated cells were resuspended with a goat anti-human mouse R-phycoerythrin-conjugated secondary antibody (ebioscience 12-4998) and incubated for 60 min in the dark. After washing with FCS buffer, the cells were fixed by addition of 3.7% formaldehyde solution. Cells were washed with PBS and subjected to flow-cytometric analysis using a FACSCanto II instrument (BD Biosciences). Fluorescence was detected at 525 nm after excitation at 405 nm. The data were analysed using FlowJo software. The Herceptin sample used for comparison in Supplementary Fig. S3 was a commercial preparation from Roche Pharma AG.

### Thermostability measurements using differential scanning fluorimetry

Differential scanning fluorimetry was used to measure DARPin unfolding by monitoring SYPRO Orange fluorescence. Purified DARPins were diluted to 5 µM in a Tris buffer (Tris 50 nM and NaCl 150 nM, pH 7.5). SYPRO Orange dye (Invitrogen) was used at a final concentration of 20×. Melting curves were recorded over a temperature range of 25–99°C (in triplicate 20 µl reaction volumes) using a real-time PCR machine (Rotor-Gene 6000, Corbett Research) with filters to excite at 460 nm and measure emission at 510 nm as the temperature continually increased (ramp rate of 1°C/min). Data were fitted using GraphPad Prism software.

## Results and discussion

### Affinity maturation of an anti-HER2 DARPin using SNAP display

The starting point for affinity maturation via SNAP display was DARPin G3-HAVD, an anti-HER2 binder previously isolated by ribosome display (*K*_D_ of 269 nM) from the N2C consensus designed ankyrin repeat library (Zahnd *et al.*, 2007a,b). We used random mutagenesis by error-prone PCR and tuned selection stringency by varying the target protein concentration to select for variants with higher affinity. As SNAP display is a fully *in vitro* system, it provides access to both of these features. Ligation- and transformation-independent assembly of the SNAP display construct via overlap PCR (Supplementary Fig. S1) allows the construction of large libraries without introducing mutations in regulatory regions. The recently published improved protocol (Houlihan *et al.*, 2014) guided the implementation of stringent washing conditions, antigen concentration modulation and DNA recovery quantification required for successful selection. Briefly, 10^9^ DNA molecules from the reassembled library were used as DNA input for selection against soluble, recombinantly expressed HER2 (extracellular domain only) immobilised on streptavidin-coated beads. Four rounds of SNAP display selections were performed, with lowering antigen concentrations from 100 to 1 nM to increase the stringency during selections (Schier *et al.*, 1996; Scott and Smith, 1990) (Table I). The polymerase used for amplification was alternated between Taq and Mutazyme at each round to introduce either on average very few [0.1 mutations per kb per cycle for Taq (Tindall and Kunkel, 1988)] or two amino acid mutations per protein (for Mutazyme). To monitor enrichment, DNA recovery (as measured by qPCR) was compared between selections performed in the presence and absence of ligand (Table I).

**Table I.** Selection conditions over four rounds of selection using SNAP display

Round	Antigen concentration (nM)	Polymerase	Recovery of DNA^a^	DNA recovery—ratio positive: negative^b^	Proportion of binders^c^ amongst recovered clones (%)
1	100	Mutazyme	1.2 × 10^6^	46	n.d
2	10	Taq	4.4 × 10^5^	37	5
3	1	Mutazyme	6.7 × 10^5^	74	n.d
4	1	Taq	2.5 × 10^6^	167	30

^a^Number of molecules measured by qPCR.

^b^Ratio refers to the recovery in selections in the presence of HER2 (positive) versus selections in the absence of HER2 (negative).

^c^Classified based on ELISA signal being greater than that of the parent G3 HAVD.

### Improved anti-HER2 DARPins selected by SNAP display reveal mutational hotspots

**Fig. 2:**
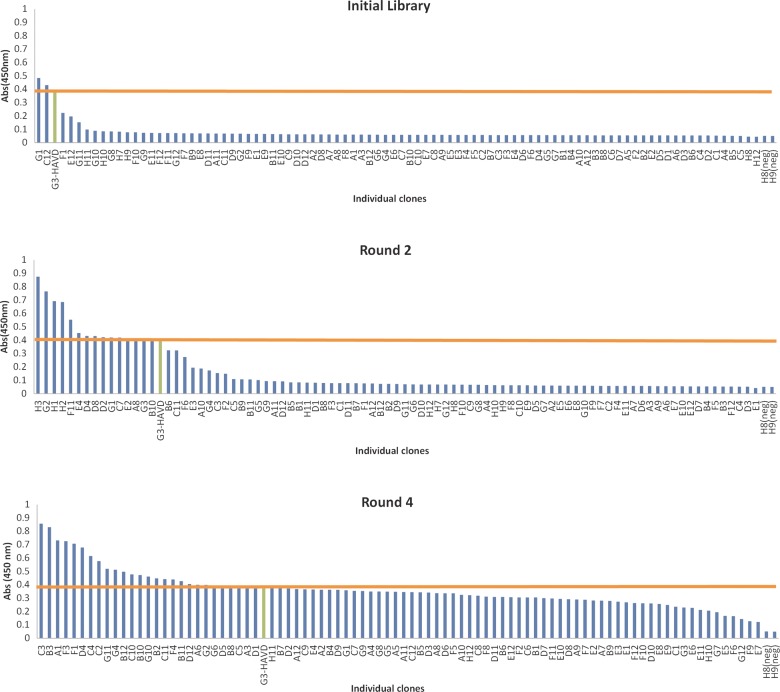
Primary screen of DARPins selected against HER2 using SNAP display. Four rounds of affinity selections were performed and 90 DARPins from the initial library, second and fourth round were tested for binding in a crude extract ELISA. Binding of DARPins to HER2 was detected using an HRP-conjugated antibody. The orange line indicates the ELISA signal of DARPin G3-HAVD in each screen. Over 90% of the clones screened gave a signal >5-fold over background while ∼30% of the selected DARPins showed greater binding signals compared with the parent DARPin G3-HAVD.

**Fig. 3:**
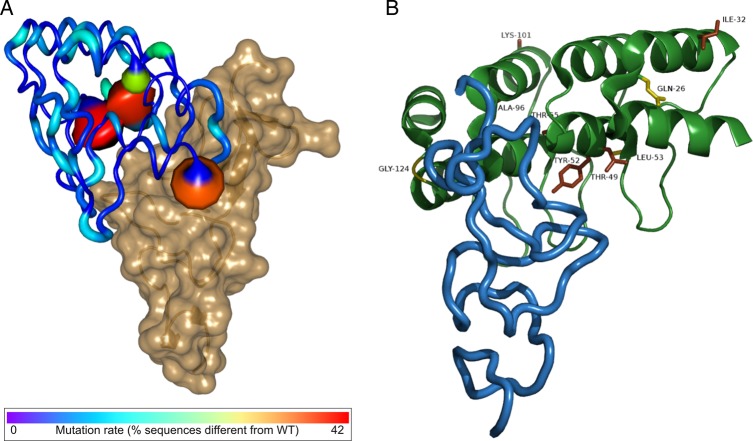
(**A**) The frequency of mutations measured in the sequenced DARPin mutants is plotted onto the structure of DARPin H10-2-G3 (shown as putty cartoon): frequently mutated positions are colour-coded and rendered with backbone thickness proportional to mutation rate. Fully conserved positions are shown in dark blue. The position that accumulated ∼42% of sequence diversity, H52, is shown in red. The positions most frequently mutated are in proximity of the binding interface. Overall, positions mutated in greater than 10% of the sequenced population are all located in framework positions in the designed N2C DARPin library. Mutations do, however, map also on framework positions away from the binding interface. The interacting domain of HER2 is represented as a semi-transparent sand-coloured surface. (**B**) The published X-ray structure of DARPin H10-2-G3 (green) in complex with HER2 (blue) (Jost *et al.*, 2013) was used to visualise the likely locations of the mutations obtained in DARPin F1. DARPin H10-2-G3 was affinity matured from the same parent clone (DARPin G3 HAVD) as the DARPins evolved by SNAP display. Mutations that contribute to binding are coloured brown while neutral mutations are coloured yellow (see Table III). Figures were prepared with Pymol based on PDB 4HRN (Jost *et al.*, 2013).

To determine the number of bona fide binders, 96 individual colonies from the initial library, round 2 and 4 outputs were separately cloned, expressed as soluble DARPins in M15 cells and ELISA screened in crude extract for binding to HER2. The initial library contained 2% clones with similar binding signal as the parent clone, while this number rose to 72% in round 4 (a 30-fold increase; Fig. 2). In round 4, 30% of the clones gave ELISA signals greater than the original DARPin, consistent with ribosome (Hanes *et al.*, 1998) and phage display (Brockmann *et al.*, 2011) outputs. The increase in hits suggests that SNAP display is capable of isolating improved HER2-binding DARPins from large epPCR-generated libraries. Sequence analysis of the DARPins that gave the highest signals in the crude extract ELISA (Fig. 2) indicated that selected clones had between two and nine amino acid changes scattered across the entire sequence compared with the parent DARPin (Supplementary Fig. S2). The majority of mutations are found in framework positions while only four (<10%) are found in originally randomised positions. Mutations cluster in the first helix of the first repeat and C-cap indicating these as potential binding hotspots (as confirmed by the mutational analysis of the top 60 isolated DARPins, Fig. 3A). Two positions in the first repeat, His52 and Ala55, are mutated in over half of the selected DARPins, indicating that optimisation of these positions is important for improving affinity. The mutation H52Y was also selected during evolution of the same parent clone using ribosome display and was found to directly interact with HER2 (Jost *et al.*, 2013). Isolation of different DARPins with distinct mutations indicates that there are multiple solutions/trajectories to improve the affinity of DARPin G3-HAVD for HER2. However, Fig. 3 shows that the majority of the highly mutated positions (and all those accumulating more than 10% variability) fall outside the originally randomised positions in the designed N2C library, and therefore epPCR was required throughout the selection process in order to acquire framework mutations.

### The high affinity of isolated DARPins is driven by improved *k*_on_ values

The 10 binders with strongest signals in the crude extract ELISA were affinity purified. To determine the affinities of the selected clones, purified proteins were used for the determination of *K*_D_ using bio-layer interferometry (Abdiche *et al.*, 2008). All selected variants had improved binding affinities for HER2 compared with DARPin G3-HAVD (Table II). Four of the binders had affinities in the low- to sub-nanomolar range, and the remaining variants had *K*_D_ values <100 nM. The association rate was the parameter most improved (100- to 1000-fold) across the panel of DARPins compared with the published *k*_on_ of DARPin G3-HAVD (2.75 × 10^3^ M^−1^ s^−1^). This observation stands in contrast to previous selections, in which affinity maturation affected the dissociation rate *k*_off_ (Yang *et al.*, 1995; Chen *et al.*, 1999; Jermutus *et al.*, 2001). While typical association rates for protein–protein interactions are on the order of 10^5^–10^6^ M^−1^ s^−1^, the parent DARPin G3-HAVD has an unusually slow *k*_on_ (2.8 × 10^3^, i.e. two orders of magnitude slower; Table II). The most improved variant, DARPin F1, has an affinity three orders of magnitude greater than the parent clone mainly due to a 1000-fold faster *k*_on_ that accounts for most of the observed affinity gain.

**Table II.** Affinities and binding kinetics of the selected HER2-binding DARPins^a^

DARPin	Mutations	*k*_on_(M^−1^ s^−1^)	*k*_off_ (s^−1^)	*K*_D_ (nM)	n-fold *K*_D_ improvement
F1	Q26E, I32V, T49A, H52Y, L53H, A55T, V96A, K101R, G124V	9.5 × 10^5^	3.7 × 10^−4^	0.39	690
F3	N36I, Y46C, H52Y, A55T, D122G	6.3 × 10^5^	1.2 × 10^−3^	1.9 ± 0.03	140
A1	A22V, D27G, A55T, V76A, K111R, D122G	2.8 × 10^6^	1.1 × 10^−2^	3.8 ± 0.09	70
C3	H52Y, A55T, A56T, E61G, K114N	1.4 × 10^6^	6.6 × 10^−3^	4.0 ± 0.05	58
D4	G25R, A55T, G124V	4.2 × 10^5^	6.7 × 10^−3^	15 ± 0.12	17
C4	E20G, N36D, H52Q, A88T, V106A, T115A, E126G	8.3 × 10^5^	5.3 × 10^−2^	64 ± 2.0	4
B3	G37D, H52Q, V76M, A104V, D122G,	1.2 × 10^5^	8.7 × 10^−3^	72 ± 0.28	4
G11	L66M, H102Y, D1104, D127V	7.1 × 10^5^	6.1 × 10^−2^	86 ± 0.26	3
C2	N36Y, N41D, E61G, L93H, V96A, K101R, G124V	2.8 × 10^5^	2.7 × 10^−2^	98 ± 0.23	3
Parent G3-HAVD^b^		2.8 × 10^3^	7.4 × 10^−4^	269	–
Trastuzumab^c^		7 × 10^5^	4 × 10^−4^	0.5	–

^a^The data were evaluated with a global kinetic fit. Measurements were performed in PBS buffer supplemented with 0.01% BSA and 0.01% Tween 20 (pH 7.4).

^b^These are published descriptors of binding as measured by BIAcore for parent G3-HAVD (Zahnd *et al.*, 2007a,b) and ^c^Trastuzumab (Bostrom *et al.*, 2011).

### Isolated DARPins bind selectively to HER2 *in vitro* and on cells

**Fig. 4:**
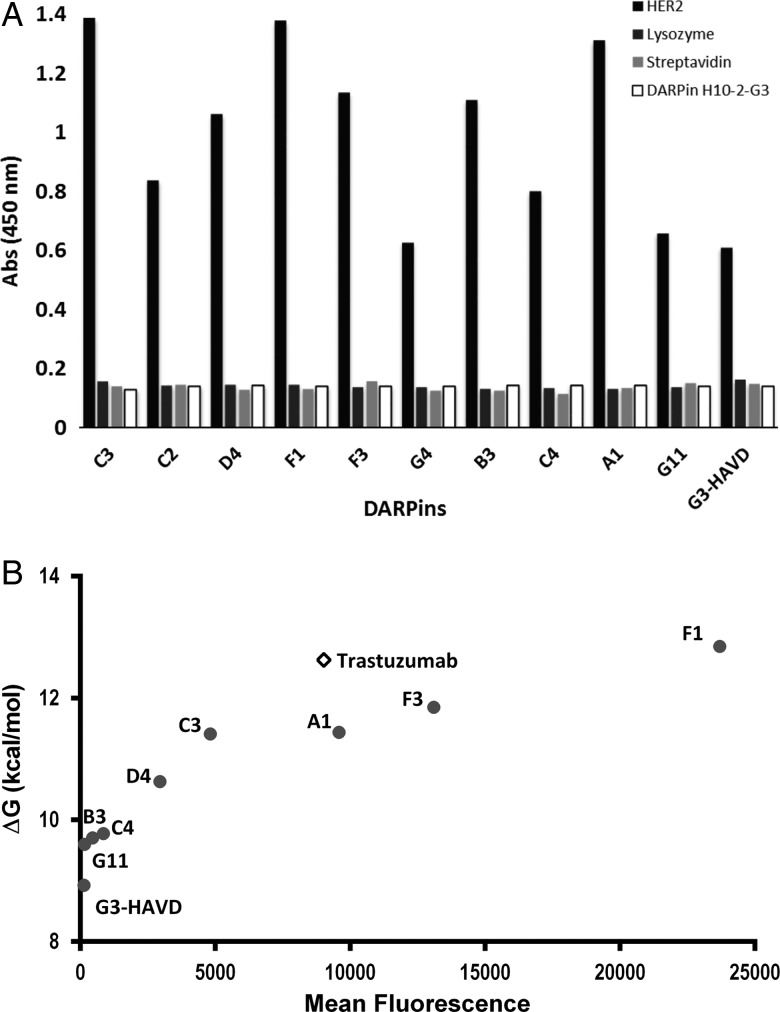
(**A**) ELISA analysis of selected DARPins. The top 10 DARPins (10 μg/ml of purified DARPin) were analysed for binding to immobilised HER2. All selected DARPins from SNAP display outputs gave a greater binding signal than DARPin G3-HAVD. All evolved DARPins were also analysed for their specificity in binding. No binding was observed to Streptavidin (which was used to capture biotinylated HER2 during selections) or lysozyme indicating the selected DARPins do not non-specifically bind to other proteins. Competition for binding of selected DARPins to HER2 with DARPin H10-2-G3 (10-fold excess DARPin H10-2-G3 over selected DARPins) showed that each selected DARPin competed for the same epitope as DARPin H10-2-G3. (**B**) Correlation between the observed fluorescence values of cell populations and the reciprocal of their binding affinity shows a correlation between the two properties. Most notably, Trastuzumab appears to have a significantly lower affinity on cells, probably as a result of the different binding mode and epitope the antibody binds to on HER2.

As *in vitro* selections are typically performed against an immobilised recombinant target in isolation, there is a risk that non-specific interactions are preferred, and that binders fail to interact when exposed to a target on a complex cellular surface. To exclude that non-specific binding during the fully *in vitro* procedure could affect the specificity of isolated DARPins, and thus compromise the use of SNAP display for the development of new therapeutics, specificity was tested both *in vitro* and on mammalian cells overexpressing HER2. Isolated DARPins were first tested in soluble ELISA against streptavidin (the binding functionality used to capture HER2 on beads during the panning procedure) as well as lysozyme (a highly charged globular protein known to provide an easy docking surface for binding). As shown in Fig. 4A, no binding to either of these targets was detected. However, when selected DARPins were incubated in the presence of an excess of the published anti-HER2 DARPin H10-2-G3 (Jost *et al.*, 2013), binding to HER2 was inhibited. This observation suggests that all SNAP display evolved DARPins compete, at least *in vitro*, for the same epitope on HER2, and that the binding regions overlap with that of DARPin H10-2-G3. To assess whether the high *in vitro* affinity could be exploited for therapeutic use, the binding performance was evaluated *in vivo* by a cell-based assay with SK-BR-3, an HER2-positive breast cancer cell line reported to express 10^6^ HER2 molecules per cell. All evolved DARPins showed specific binding to HER2 on cells, while no binding was observed for DARPin HDKV, a DARPin known not to bind to HER2 (Supplementary Fig. S3). The fluorescence intensities correlate with the affinities of the evolved panel of binders measured *in vitro* with soluble HER2 (Fig. 4B). Two evolved DARPins (F3 and A1) with *in vitro* affinities lower than Trastuzumab (Table II) showed higher fluorescence intensities. While Trastuzumab binds to an epitope on domain IV of HER2 located close to the membrane (Cho *et al.*, 2003), the panel of SNAP display evolved DARPins bind to an epitope located further from the membrane (Jost *et al.*, 2013). However, the use of different techniques to attach fluorescent labels means that the number of fluorophores per protein molecule is not normalised and the intensity readout may not report on affinity. The accessibility of the different epitopes may explain the observed difference in binding on cells. Although SNAP display selections were performed entirely *in vitro* using recombinant HER2, the isolated variants are able to recognise and bind their target when expressed in a complex cellular milieu.

### Evidence for a stability/function trade-off during affinity maturation

**Fig. 5:**
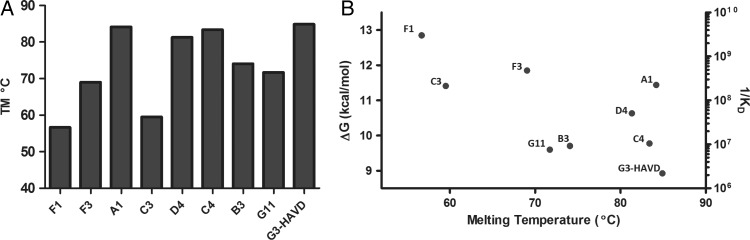
Thermal stability analysis of the selected DARPins. (**A**) Melting temperatures of the selected DARPins were measured by differential scanning fluorimetry (plotted based on increase in affinity going from right to left). Measurements were performed in Tris buffer and repeated twice for all mutants. (**B**) Correlation between melting temperature and affinity of selected DARPins. An order of magnitude in binding strength leads to a ∼8°C loss in stability (correlation coefficient *r* = 0.43; fit not shown).

In order to analyse how the mutational load imposed on the DARPin structure is productively used for creating binding interactions, the thermostabilities of the parent and evolved DARPins were measured using differential scanning fluorimetry (Niesen *et al.*, 2007; Gatti-Lafranconi *et al.*, 2013). Directed evolution studies on enzymes have revealed a trade-off between stability and function along evolutionary trajectories (Tokuriki *et al.*, 2008; Thomas *et al.*, 2010). The thermal stabilities of DARPin variants tend to be decreased compared with the parent (Fig. 5A). Among the selected mutants that show improved binding, five are destabilised by greater than 10 degrees (DARPin F1, C3, F3, G11 and B3), while three other DARPins (A1, D4 and C4) maintain high levels of stability. The clone with greatest affinity, DARPin F1, also lost the largest amount of thermal stability (30°C). A correlation between affinity and stability for DARPins selected by SNAP display (Fig. 5B) is dominated by the destabilisation of three of the four most improved mutants, but overall suggests a trade-off between these parameters. An order of magnitude in binding strength leads to an ∼8°C loss in stability (albeit with a moderate correlation coefficient *r* = 0.43). A similar trend is observed when the number of mutations is plotted against *T*_m_ (slope: −0.1188 ± 0.07773 kcal/°C). There is extensive scatter in these plots, presumably reflecting the idiosyncratic effects of individual mutations, but our results are in line with other examples of stability loss upon gain of improved binding and extend previous analyses of stability/function trade-offs in protein binders (Hackel *et al.*, 2008; Karanicolas *et al.*, 2011). Whilst stabilising mutations have been found in antibodies evolved during an immune response to compensate for destabilising binding mutations (Wang *et al.*, 2013), *in vitro* evolved DARPins show a different behaviour. This may be due to the high stability of DARPins compared with antibodies and the secondary selection for stability during *in vitro* evolution compared with natural evolution.

### Analysis of the effects of individual mutations accumulated in DARPin F1 on binding affinity and thermal stability

DARPin F1 was chosen for further study because it showed the greatest affinity improvement and is the least thermostable of the evolved clones. DARPin F1 shares three mutations (H52Y, A55T, V96A) with DARPin H10-2-G3 that has a *K*_D_ in the picomolar range for HER2 (Zahnd *et al.*, 2007a,b). DARPin F1 also acquired six other mutations (Q26E, I32V, L53H, T49A, K101R and G124V) throughout the four rounds of selection (Fig. 3B). As DARPin F1 competes with DARPin H10-2-G3 for binding to HER2 (Fig. 4A), we assume that they bind to the same epitope and thus used the crystal structure of the bound complex to guide our analysis (Jost *et al.*, 2013). To test the role of each mutation accumulated by DARPin F1 for affinity towards HER2, each mutation was individually reverted to the original amino acid present in DARPin G3-HAVD and their contribution to binding in DARPin F1 assessed (Table III). Three mutations were functionally neutral (G124V, T49A and Q26E) while six others (L53H, I32V, K101R, H52Y, A55T and V96A) contributed almost entirely to binding only (as indicated by the decrease in affinity when reverted to their original residues in the parent DARPin, see Supplementary Table S1). Stability loss upon gain of function is observed for DARPin F1: its melting temperature is almost 30°C lower than that of the parent DARPin (*T*_m_ = 56.7°C versus *T*_m_ = 85.9°C, Fig. 5). The effects of these nine individual mutations in DARPin F1 are not simply additive: they add up to an increase in *T*_m_ of ∼11°C when individually reverted, falling short of the observed 30°C of destabilisation when combined suggesting negative epistasis between destabilising mutations (Olson *et al.*, 2014).

**Table III.** Position, contribution to stability and affinity of individual DARPin F1 mutations^a^

DARPin F1 residue reversion	Residue position^b^	*K*_D_ increase (*n*-fold)	Δ*T*_m_ (°C)	Effect on binding/stability^c^	
Q26E	Framework	1.8	6.9	**≈**	/ **+**
I32V	Framework	243	−2.0	**+**	/ **−**
T49A	Framework	0.8	−4.5	**≈**	/ **−**
H52Y	Framework	160	−6.1	**+**	/ **−**
L53H	Framework	7.5	6.8	**+**	/ **+**
A55T	Framework	52.5	6.2	**+**	/ **+**
V96A	Framework	57.5	−1.5	**+**	/ **−**
K101R	Framework	15.5	3.9	**+**	/ **+**
G124V	Framework	–	0.9	**≈**	/ **+**

^a^See Supplementary Table S1 for raw values.

^b^Positions as defined in Zahnd et al. (2007a,b).

^c^+ indicates an increase in binding and/or affinity. ≈ indicates a mutation is neutral with respect to binding while – signifies a mutation is destabilising.

Investigation of the thermal stability of individual revertants revealed that mutations which had the greatest influence on binding affinity (H52Y, I32V and V96A) also influenced thermal stability to the largest extent (when reverted, mutants had melting temperatures 4–6°C greater than DARPin F1, Table III)). Other functional mutations that contribute to binding to a lesser extent (L53H and K101R) decreased thermal stability suggesting their role in DARPin F1 involves both improving affinity and stabilising the protein (and thus counteracting the functionally destabilising mutations). Functionally silent mutations Q26E and G124V both have stabilising effects, while, surprisingly, T49A destabilises the protein by 4°C, showing the lack of a stringent selection for stability (Supplementary Fig. S4). To investigate the contribution functional mutations have on DARPin F1 thermostability, the mutations that individually do not significantly affect *K*_D_ (Q26E, T49A and G124V), but have contrasting effects on thermal stability, have been reverted to give the mutant F1* containing only mutations that contribute to binding. This mutant showed a moderate (∼3-fold) decrease in affinity towards HER2, while the melting temperature dropped a further 5°C (Table III). This observation is consistent with the trade-off hypothesis between thermal stability and binding affinity as it indicates that functionally relevant mutations had an overall destabilising effect. The functionally neutral but destabilising mutation T49A highlights the weak selection pressure for stability and the highly stable nature of DARPins that allows them to tolerate such destabilising mutations, yet sustain function. Thus, while compensatory stabilising mutations were observed, stability was not restored to parent DARPin levels, which we hypothesise is due to the selection pressure for stability being secondary to affinity, provided a library member is above the minimum thermodynamic stability threshold.

## Conclusions

### SNAP display is a powerful tool for affinity maturation

The data presented here constitute the first example of the use of SNAP display for molecular evolution of a binding protein. Starting from a known binder with high nanomolar affinity for HER2, we generated diversity by epPCR, selected for binding with decreasing concentrations of antigen over multiple rounds of *in vitro* selections by SNAP display and successfully isolated sub-nanomolar-binding anti-HER2 DARPins. Screening the outputs from the SNAP display selection rounds evolved a number of different binders that bound HER2 with greater affinity than the parent clone. The evolved DARPin with highest affinity has a K_D_ almost 700-fold greater than its progenitor. The improved panel of DARPins validate SNAP display as a platform for directed evolution of protein binders. Compared to the published ribosome display affinity maturation against HER2 (six rounds including off-rate selections, (Zahnd *et al.*, 2007a,b)), SNAP display required only four rounds of 1 h soluble selections to isolate proteins with high affinity. We speculate that the excess of proteins (∼10^5^ molecules) uncoupled to the genotype that is produced in each DNA template containing droplet (Houlihan *et al.*, 2014) leads to highly competitive binding conditions during panning and may be an unexpected advantage of affinity selections using SNAP display, given its smaller library size (up to 10^14^ in ribosome or mRNA display versus up to 10^9^ in display methods that rely on droplet compartmentalisation). Other display technologies that rely on *in vitro* compartmentalisation have been utilised for directed evolution of binding proteins, e.g. zinc finger binding proteins (Sepp and Choo, 2005), Fab fragments (Sumida *et al.*, 2012) and src homology 3 (SH3) domains (Bertschinger *et al.*, 2007) with affinities in the low nanomolar range. The intrinsic stability of DNA (compared with RNA) and the covalent link between genotype and phenotype in SNAP display should facilitate approaches that rely on further modification of the displayed protein (Heinis *et al.*, 2009; Josephson *et al.*, 2014; Passioura *et al.*, 2014) and in applications beyond, where display constructs are part of more complex assemblies (Gu *et al.*, 2014). SNAP display is capable of selecting binders in the sub-nanomolar range and represents a powerful droplet-based method for directed evolution of protein binders.

### Framework mutations arise during affinity maturation and contribute to binding

Although repeat proteins have clearly distinguished binding interfaces that display sets of loops making them exceptionally amenable to rational redesign, the majority of mutations (47 of 52) in the panel of affinity matured DARPins are not located in these contact loops, but instead in framework positions. This is due to the library synthesis by epPCR that permitted mutations throughout the entire DARPin. Mutations accumulated by the highest affinity variant, DARPin F1, are in framework positions located away from the binding interface. While antibodies typically accumulate somatic mutations in the CDR loops that are responsible for improved affinities, mutations in the framework region imply that subtle structural changes may also lead to affinity increases (Klein *et al.*, 2013). Intra- and inter-repeat interactions found in DARPins, including an extensive hydrogen bonding network are thought to contribute to the extreme stability of these molecules (Kohl *et al.*, 2003). The side chain of framework residue His52 forms intra-repeat hydrogen bond with Thr49 and also with the carbonyl oxygen of residue 81 (a randomised position in the β-turn of the third repeat). The crystal structure of DARPin H10-2-G3 (which also contains the H52Y mutation found in some SNAP display evolved DARPins) shows that H52Y disrupts this hydrogen bonding pattern leading to a 13° rotation of the C-cap and an adjacent randomised repeat compared with an unmutated DARPin (Zahnd *et al.*, 2007a,b). As DARPins are designed to be extremely stable and thus somewhat rigid, structural rearrangements may be required to increase affinity, which may explain the accumulation of mutations in framework positions, thus corroborating our randomisation approach combined with SNAP display selections.

### The stability of the DARPin structure accommodates a high mutational load

Thermal denaturation analysis showed that the evolved panel of SNAP display-selected DARPins are less thermostable than the parent DARPin G3-HAVD, consistent with other studies of protein binders that suggested trade-off between stability and function throughout evolutionary trajectories (Foit *et al.*, 2009; Hackel *et al.*, 2008; Karanicolas *et al.*, 2011). DARPin G3-HAVD binds HER2 with low affinity, whilst retaining thermostability levels in the range reported for members of the naïve library (Zahnd *et al.*, 2006), DARPins evolved by SNAP display had an affinity gain of three orders of magnitude in some cases, yet simultaneously lost up to 30°C of thermostability. We find that DARPin F1 acquired both functional destabilising mutations (H52Y, I32V and V96A) and compensatory stabilising mutations (Q26E and G124V) throughout the affinity maturation process *in vitro*. However, compensatory mutations failed to restore stability to parent levels. The selection pressure exerted *in vitro* throughout SNAP display selections is therefore directed towards affinity optimisation, while protein stability is secondary. The high thermal stability of DARPins appears to be key for the success of selections, because a large mutational load can be accommodated. In this way, the library can be harvested primarily for the criterion of binding, before a co-selection for stability constricts the effective library size. High thermodynamic stability has been shown elsewhere to increase the mutational robustness and evolvability of proteins (Bloom *et al.*, 2005; Bloom *et al.*, 2006; Tokuriki and Tawfik, 2009; Tracewell and Arnold, 2009) and DARPins benefit from the ability to readily accommodate a large number of mutations, emphasising the utility of the DARPin consensus design for protein evolution. Our selection strategy stands in contrast to evolution of repeat proteins in Nature. The modular nature of repeat proteins has led to their evolution through repeat expansion and homologous recombination without significant impact on structural stability. Consensus design of DARPins has successfully produced exceptionally stable proteins due to extensive intra- and inter-repeat interactions (Binz *et al.*, 2003). Therefore, mutations in the framework are likely to disrupt such interactions and decrease overall stability. Nonetheless, even after stability loss upon improved function, evolved DARPins maintain high levels of thermostability with the majority exhibiting melting temperatures > 60°C and are therefore sufficiently stable for applications in diagnostics and therapeutics.

These results illustrate SNAP display as a powerful method for affinity maturation of DARPins, which should enable the rapid development of high-affinity proteins targeted to relevant therapeutic targets and establish it as a part of the protein engineering toolkit.

## Authors’ contributions

G.H. performed experiments, G.H., P.G.-L., D.L. and F.H. designed and analysed experiments, F.H. and D.L. directed research and G.H., P.G.-L., D.L. and F.H. wrote the paper.

## Supplementary data

Supplementary data is available at *PEDS* online.

## Funding

G.H. was supported by a CASE studentship from the Engineering and Physical Sciences Research Council and MedImmune and the Marie-Curie Research Training Network ENEFP. F.H. is a starting investigator of the European Research Council. Funding to pay the Open Access publication charges for this article was provided by the Engineering and Physical Sciences Research Council.

## Supplementary Material

Supplementary Data
