# Supplementary material for: Directed evolution of anti-HER2 DARPins by SNAP display reveals stability/function trade-offs in the selection process
Source: Protein Eng Des Sel. 2015 Jun 30;28(9):269–79. doi: 10.1093/protein/gzv029 (PMC4550541; doi:10.1093/protein/gzv029)
Supplement: Supplementary Data [file supp_28_9_269__index.html]

Directed evolution of anti-HER2 DARPins by SNAP display reveals stability/function trade-offs in the selection process — Supplementary Data 

# Directed evolution of anti-HER2 DARPins by SNAP display reveals stability/function trade-offs in the selection process

## Supplementary Data

Supplementary Data

- Supplementary Data - Pdf file
