# Supplementary material for: Directed evolution of anti-HER2 DARPins by SNAP display reveals stability/function trade-offs in the selection process
Source: Protein Eng Des Sel. 2015 Jun 30;28(9):269–79. doi: 10.1093/protein/gzv029 (PMC4550541; doi:10.1093/protein/gzv029)
Supplement: Supplementary Data [file supp_gzv029_gzv029supp.pdf]

Directed evolution of anti-HER2 DARPins by SNAP display reveals stability/function trade-offs in the selection process.

Gillian Houlihan[1,2], Pietro Gatti-Lafranconi[1], David Lowe[2] and Florian Hollfelder[1*].
[1] Department of Biochemistry, University of Cambridge, 80 Tennis Court Road, Cambridge CB2 1GA, UK.
[2] MedImmune Ltd, Milstein Building, Granta Park, Cambridge, CB1 6GH, UK.

Supplementary Information

Figure S1

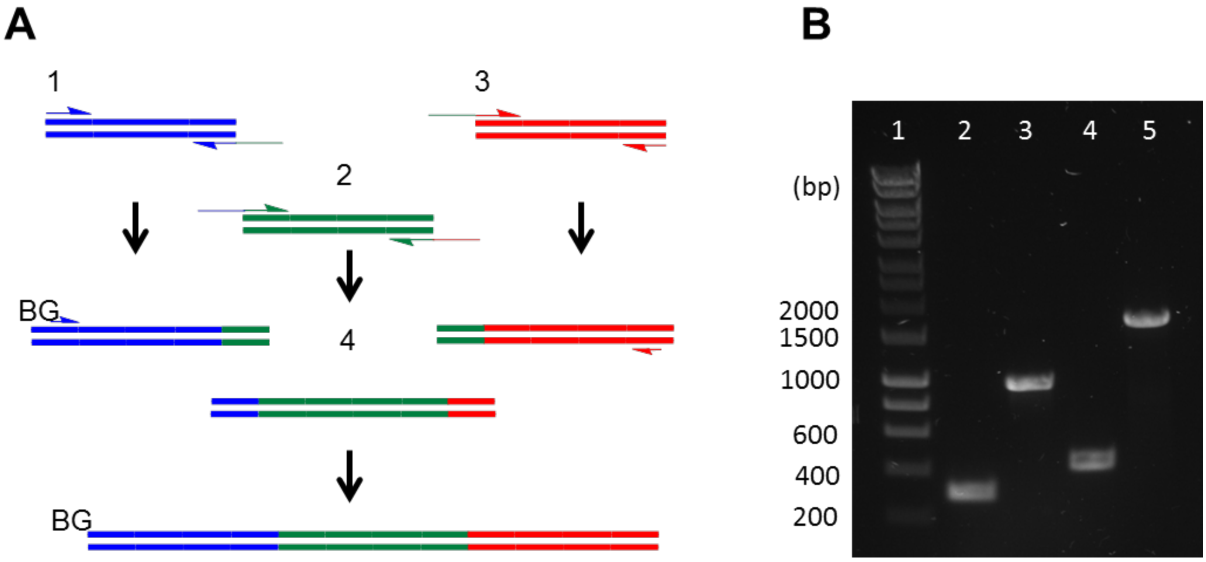

Figure S1: A) Construction of a DARPin library for SNAP display. Two PCR amplification steps were required to generate a full length DNA fragment encoding the regulatory elements required for *in vitro* expression consisting of AGT connected with a DARPin library member through a short eight amino acid linker. Firstly, DARPin-G3-HAVD was used as a template for error prone PCR (2) while the 5' untranslated region combined with AGT (1) and the 3' untranslated region (3) were amplified in separate reactions. In the second step, all three DNA fragments were used in an overlap PCR step (4) to generate full length DNA templates suitable for SNAP display. B) Agarose gel (1%) showing DNA fragments from separate PCR steps. Lane 1: marker, Lane 2: 3' UTR (266 bp), Lane 3: 5' UTR-AGT (944 bp), Lane 4: error prone DARPin library (426 bp), Lane 5: full length reassembled construct (1599 bp).

Figure S2

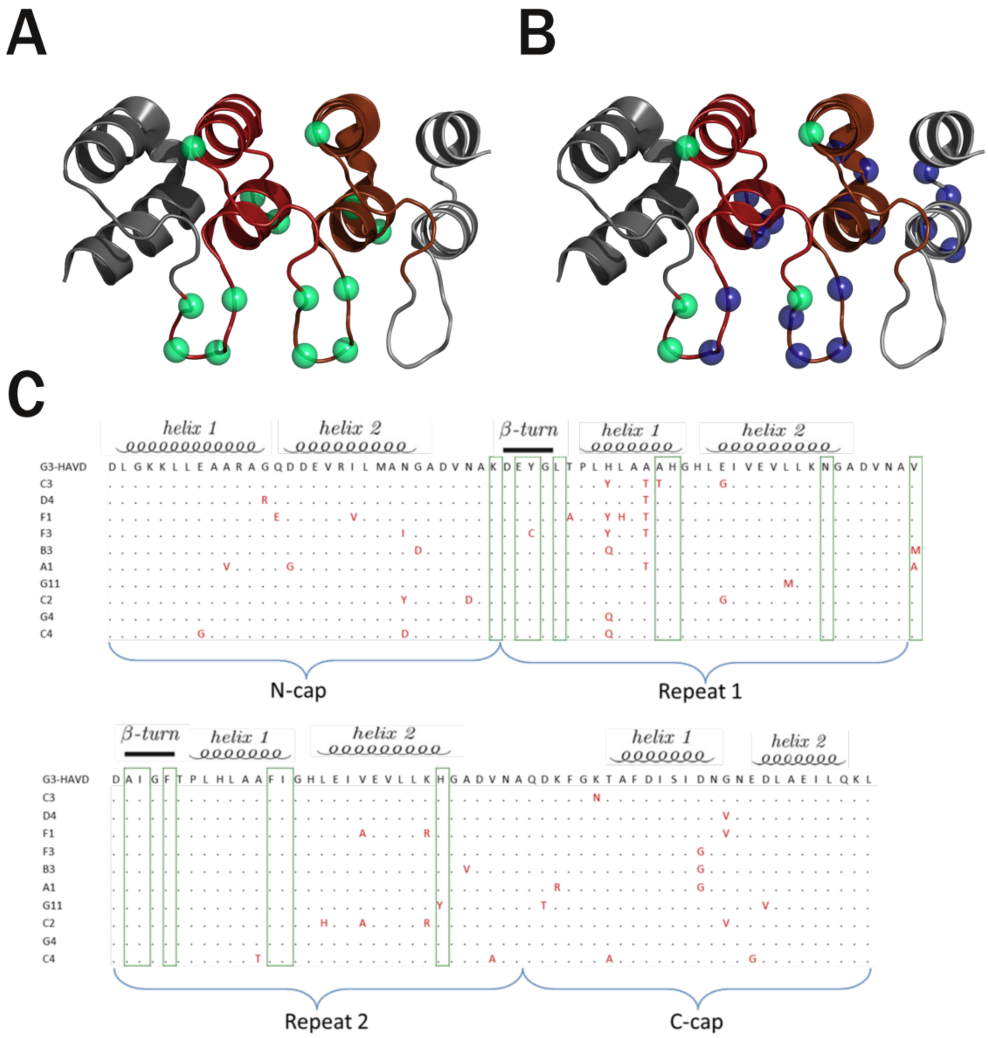

Figure S2:. Location of Mutations Arising in the Directed Evolution of DARPin H10-2-G3. (**A**) Structure of DARPin H10-2-G3 (PDB 4HRN) showing the N- and C-caps (dark and light grey, respectively), and the two repeats (red and brown) containing the randomised position (green spheres). (**B**) Same figure as panel A where the cα of all amino acids within 4 Å of HER2 are shown as blue spheres. The comparison reveals how randomised position represent a subset only of the amino acids potentially involved in the binding interaction. (**C**) Sequence alignment of the affinity matured DARPins containing two randomised repeats that were selected after four rounds of SNAP display with the parent DARPin G3-HAVD. Positions that were randomized in the original consensus design DARPin library (Binz et al., 2004) are highlighted by the green boxes. All mutations that occurred throughout the SNAP display selections are indicated in red. Secondary structure elements and the boundaries of the different DARPin repeats/units are indicated either above or below the sequence alignment, respectively.

Figure S3

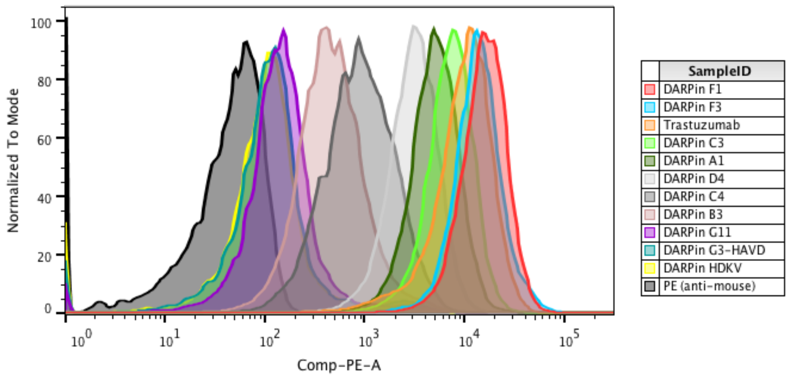

Figure S3: Flow cytometric analysis of DARPins binding to a human cell line overexpressing HER2. Purified DARPins were incubated with the HER2 expressing SKBR-3 cell line. Binding was detected using an anti-His antibody and a secondary red fluorescent PE (R-rhycoerythrin)-conjugated antibody. The plot shows the cell count as a function of the measured red fluorescence. No detectable binding of DARPin HDKV (a nonbinding control) or anti-mouse antibodies to SKBR-3 cells was observed.

Figure S4

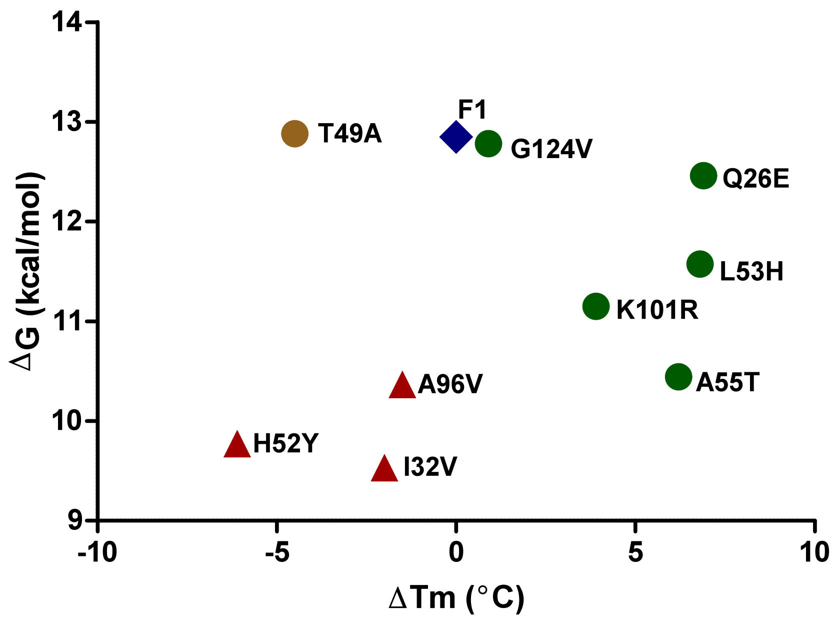

Figure S4: Characterisation of DARPin F1 reversion mutants' affinities and stabilities. The mutations accumulated by DARPin F1 throughout the affinity maturation process were reverted to the parent amino acid residues and their binding to HER2 measured using bio-layer interferometry. The thermal stability of each mutant was measured using differential scanning fluorimetry in the absence of HER2. Red colouring indicates mutations that decrease affinity and stability when reverted to the parental amino acids (in DARPin G3-HAVD), green indicates stabilising mutations with overall improved binding brown indicates a destabilising, but functionally neutral mutation.

Table S1. Binding kinetics of DARPin F1 revertants.

**DARPin**	K_D (nM)	k_{on} ($M^{-1}s^{-1}/10^5$)	k_{off} ($s^{-1}/10^{-3}$)	T_m (°C)
F1	0.35	33	0.12	56.7
F1* [a]	0.99	24	2.3	51.8
F1 Q26E revertant	0.7	18	1.2	49.8
F1 I32V revertant	97	1.1	10	58.7
F1 T49A revertant	0.33	17	0.55	61.2
F1 H52Y revertant	64	3.8	25	62.8
F1 L53H revertant	3.0	6.9	2.1	49.9
F1 A55T revertant	21	12	25	50.5
F1 V96A revertant	23	3.9	9.1	58.2
F1 K101R revertant	6.2	5.9	3.6	52.8
F1 G124V revertant	0.39	9.5	0.37	55.8

[a] The mutant F1* contains only the mutations that contribute to binding (namely I32V, H52Y, L53H, A55T, V96A AND K101R)
